# Quality of life of adolescent idiopathic scoliosis patients under brace treatment: a brief communication of literature review

**DOI:** 10.1007/s11136-020-02671-7

**Published:** 2020-10-24

**Authors:** Huan Wang, Daniel Tetteroo, J. J. Chris Arts, Panos Markopoulos, Keita Ito

**Affiliations:** 1 Department of Industrial Design, Eindhoven University of Technology, Eindhoven, Netherlands; 2 Department of Biomedical Engineering, Eindhoven University of Technology, Eindhoven, Netherlands; 3 Department of Orthopaedic Surgery, Maastricht University Medical Centre (MUMC+), Maastricht, Netherlands

**Keywords:** Adolescent idiopathic scoliosis, Brace treatment, Quality of life, Self-image

## Abstract

**Purpose:**

To identify the life domains that are most frequently reported to be affected in scoliosis patients undergoing brace treatment.

**Methods:**

A search within the PubMed database was conducted and a total of 60 publications were selected. We classified the studies based on the methods used to measure patients’ quality of life (QoL) and categorized the life domains reported to be affected.

**Results:**

Self-image/body configuration was the most reported affected domain of patients’ QoL, identified in 32 papers, whilst mental health/stress was the second most reported affected domain. Mental health was identified in 11 papers, and 11 papers using the BSSQ questionnaire reported medium stress amongst their participants. Vitality was the third most reported affected domain, identified in 12 papers.

**Conclusions:**

Our review indicates that scoliotic adolescents treated with bracing suffer in their quality of life most from psychological burdens. To improve these patients’ life quality, more attention should be focussed on supporting their mental health.

## Introduction

Adolescent idiopathic scoliosis (AIS) is defined as a three-dimensional spinal deformity with a twisting curvature that happens in juveniles of the age from 10 to 20 with no known specific aetiology. Treatment and classification guidelines have been established by the International Scientific Society on Scoliosis Orthopaedic and Rehabilitation (SOSORT) [1–3]. The SOSORT guidelines recommend observation, exercise, brace treatment or surgical treatment based on the severity of curvature.

The efficacy of brace treatment depends on both the quantity (compliance), which is defined as the percentage of actual brace-wearing time relative to the prescribed bracing time [4], and the quality (strap tightness) of brace usage [5]. The quantity of brace usage depends on patients’ own initiative in wearing the brace, where patients tend to be non-complaint reducing wearing time because of physical and psychological issues [4, 6]. This is important because the risk for curve progression and surgery are reduced in patients with good brace compliance [7].

Many factors have been reported to impact the QoL of AIS brace wearers, e.g. back pain, appearance configuration, and mental health [8, 9]. Improving QoL might increase treatment compliance amongst scoliotic brace wearers, positively impacting the treatment quantity. However, in order to effectively improve the QoL of scoliotic brace wearers, we need to know which factors most prominently impact their QoL. Different methods have been applied in measuring the QoL of AIS patients, including standardized (self-assessment) questionnaires and interviews. This paper aims to answer the question: *What are the most frequently reported affected domains of QoL of AIS patients under brace treatment?*

We answer this question by reviewing the literature on the QoL of AIS patients during brace treatment, and by classifying the literature into 5 groups based on the methods they use to measure patients’ QoL. Based on the reported results, and by comparing the results from papers using similar methods, we identify the most affected domains for AIS brace wearers’ QoL.

## Methods

### Search strategy and study selection

A search within the PubMed database was conducted on June 6, 2019, with the query: “adolescent idiopathic scoliosis AND brace treatment AND quality of life”. Results were not limited by publication date. Studies were excluded if they A) are review papers, B) involved AIS patients under surgical treatment and assessed their QoL, C) were published not as full-text in English.

### Data extraction and synthesis

Data were extracted from the included publications using a standardized form recording title, authors, sample size, methods, outcome measures and results.

The results of all the reviewed papers were analysed and grouped per patient reported outcome measurement questionnaire. Finally, the most affected domains were identified either based on the authors’ self-report, or if the authors did not explicitly identify the most affected domains, by selecting those domains with QoL results below a threshold value. These threshold values were selected based on the threshold values used by the authors who self-reported on most affected domains. For publications in which the authors concluded that no significant differences were found, neither within different domains of one questionnaire nor within different cohorts using the same questionnaire, we used the classification “No Significance”.

## Results

**Fig. 1:**
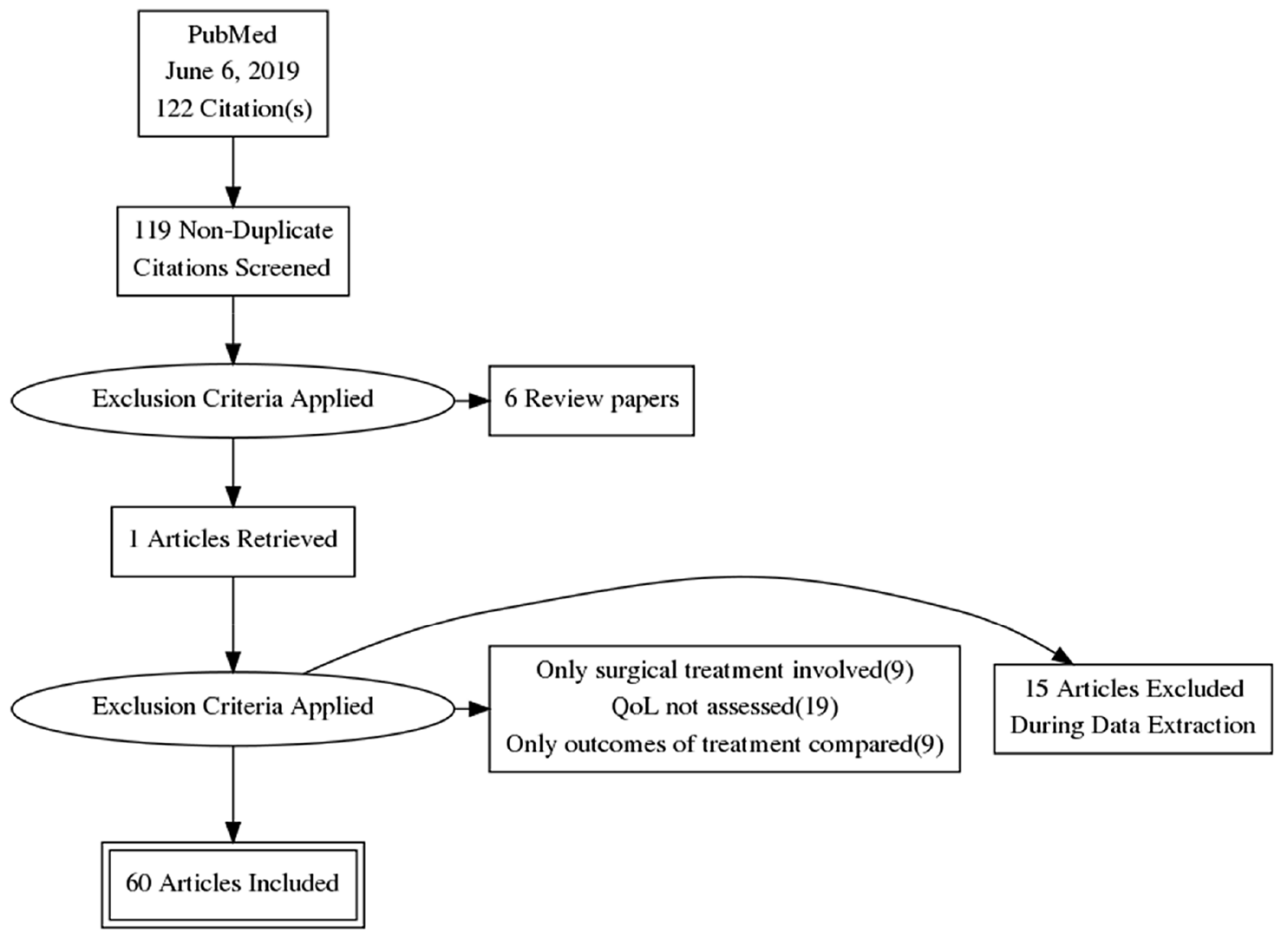
The selection flowchart of the results from the literature search

The PubMed search returned 122 papers. Publications were imported from Pubmed into Zotero^1^ and checked for duplicates. Then, titles and abstracts and potentially eligible publications were screened based on the exclusion criteria by the first author (HW). Candidates were discussed with the second author (DT) and included in the review upon mutual agreement. The articles selection process is shown in Fig. 1.

Table 1 lists the outcomes of all reviewed publications. Overall, self-image/body configuration was the most affected domain of patients’ QoL, mentioned in 32 out of 48 papers measuring self-image. Mental health was the second most affected domain mentioned in 11 out of 49 papers measuring mental health and in 11 out of 11 papers measuring psychological stress. Vitality was the third most affected domain mentioned in 12 out of 21 papers measuring vitality.

**Table 1:** An overview of the papers using different methods to measure QoL, indicating the QoL domains that were reportedly most affected by brace wearing

	References	N.(C/E)	Affected domains							
			Vitality	Self-image	Emotional function	General health perception	Physical function	School function	Bodily pain	Social activity
BrQ	Vasiliadis et al. (Greece), 2006 [10]	28								
	Kinel et al. (Poland), 2012 [11]	35	√	√	√					
	Aulisa (Italy), 2013 [12]	108	√		√	√				
	Deceuninck et al. (France), 2017 [13]	40	√	√	√					
	Gür et al. (Turkey), 2017 [14]	28	√	√		√				
	Siu Ling Chan et al. (China), 2014 [15]	42	√	√		√				
	Jong Min Lim (Korea), 2018 [16]	103	√	√	√		√	√		
	Aulisa et al., 2010 [17]	108	√		√	√				
	Elias Vasiliadis et al., 2008 [18]	32		√	√	√	√	√	√	
	Rivett et al. (South Africa), 2009 [19]	31	√	√						
	Elias Vasiliadis et al., 2006 [20]	36	√				√			
	Piantoni et al., 2018 [21]	43 female	A:56% NA:44%		A:72% NA:28%					
	In total		10	7	6	5	3	2	1	0

			BSSQ-Deformity	BSSQ-Brace
	Aulisa et al., 2010 [17]	108	–	12.6
	Michalina Zimon et al., 2018 [22]	63	18	9.5
	Kinel et al., 2012 [23]	45	15	12
	Kotwicki et al., 2007 [24]	111 female	18	9
	Misterska et al., 2009 [25]	35 female	1st evaluation:17.9 2nd evaluation:17.6	1st evaluation:11.3 2nd evaluation:10.9
BSSQ	Misterska et al., 2011 [26]	64	Urban patients:18.0 Rural patients:17.0	Urban patients:12.9 Rural patients:12.3
	Leszczewska et al., 2012 [27]	73	19	10
	Misterska et al., 2012 [28]	63 female	17.61	13.06
	Misterska et al., 2013 [29]	36 female	1st evaluation:17.7 2nd evaluation:18.0 3rd evaluation:18.1	1st evaluation:13.8 2nd evaluation:14.1 3rd evaluation:15.4
	Xu et al., 2015 [30]	86	15.3	13.4
	F. Rezaei Motlagh et al., 2018 [31]	53	15.38	12.08
	In total		Medium stress:3 papers	Medium stress:11 papers

	References	N.(C/E)	Affected domains				
			Self-image	Satisfaction	Mental health	Function activity	Pain
	﻿Aulisa et al., 2013 [12]	108	√				
SRS-22	﻿Gür et al., 2018 [14]	28	√		√		
	﻿Chan et al., 2014 [15]	42	√				
	﻿Jong Min Lim et al., 2018 [16]	103		√			
	﻿Aulisa et al., 2010 [17]	108	√				
	﻿Misterska et al., 2013 [29]	36 female	√		√	√	
	﻿F. Rezaei Motlagh et al., 2018 [31]	53	√			√	
	﻿Cheung et al., 2007 [32]	46	√			√	
	﻿Schreiber et al., 2015 [33]	50	√				
	﻿Mousavi et al., 2010 [34]	84	√				
	﻿Danielsson et al., 2012 [35]	77 female	√				
	﻿Qiu et al., 2011 [36]	54	√				
	﻿Ersen et al., 2016 [37]	64	√	√	√		
	﻿Lange et al., 2011 [38]	214	√				
	﻿Deceuninck et al., 2012 [39]	120	√				
	﻿Simony et al., 2015 [40]	73	√	√			
	﻿Yagci et al., 2018 [41]	20 female	√				
	﻿Yagci et al., 2019 [42]	30 female	√		√		
	﻿Cheung et al., 2019 [43]	652	√				
	﻿Larson et al., 2019 [44]	77	√	√			
	﻿Cheung et al., 2016 [45]	206	√	√			
	﻿Danielsson et al., 2015 [46]	197		√			
	﻿Müller et al., 2011 [47]	38		√		√	
	In total		20	7	4	4	0
	﻿Diarbakerli et al., 2018 [48]	100	No significance				
	﻿Paolucci et al., 2017 [49]	32	No significance				
	﻿Danielsson et al., 2010 [50]	459	No significance				
	﻿Bunge et al., 2007 [51]	11	No significance				
	﻿Danielsson et al., 2013 [52]	52	No significance				

	References	N.(C/E)	Affected domains							
			Physical function	Bodily pain	General health	Role limitations due to physical problems	Vitality	General mental health	Role limitations due to emotional problems	Social function
SF-36	﻿Qiu et al., 2011 [36]	54				√				
	﻿Danielsson et al., 2015 [46]	197(130/67)	√	√		√				
	﻿Danielsson et al., 2001 [53]	216(100/116)	√							
	﻿Danielsson et al., 2003 [54]	209(100/109)	√	√						
	﻿Freidel et al., 2002 [55]	146		√			√	√		
	﻿Andersen et al., 2006 [56]	484(76/408)	√		√		√			
	In total		4	3	1	2	2	1	0	0
	﻿Danielsson et al., 2012 [35]	77(37/40)	﻿No data listed, only compared with outcomes from other questionnaires, no significant differences were found							
	﻿Simony et al., 2015 [40]	73	﻿Patients got lower score in Physical Composite summary than Mental Composite summary							
	﻿Danielsson et al., 2006 [57]	202	﻿No significant difference was found in physical functioning and Physical Composite summary							

			Measuring methods	Affected domains
Other methods	﻿ ﻿Schreiber et al., 2015 [33]	50(25/25)	SAQ	Spinal appearance
	﻿ ﻿Carreon et al., 2011 [58]	1802	SAQ	Spinal appearance
	﻿ ﻿Schwieger et al., 2016 [59]	319(120/199)	SAQ	No Significance
	﻿Schwieger et al., 2017 [60]	167	SAQ	No Significance
	﻿Cheung et al., 2019 [43]	652	EQ-5D-5L	No Significance
	﻿Cheung et al., 2016 [45]	227	EQ-5D-5L	Pain
	﻿Korovessis et al., 2007 [61]	103(62/41)	QLPSD	Back flexibility
	﻿Pham et al., 2008 [62]	108(32/76)	QLPSD	Back flexibility
	﻿Weigert et al., 2006 [63]	44	SRS-24	General self-image Satisfaction
	﻿Wibmer et al., 2018 [64]	41	SRS-24	Back functions
	﻿Danielsson et al., 2003 [54]	209(100/109)	GFS	Back functions
	﻿Freidel et al., 2002 [55]	146	BFW	Self-image
	﻿Ugwonali et al., 2004 [65]	214(136/78)	CHQ	No Significance
	﻿Ugwonali et al., 2004 [65]	214(136/78)	PODCI	No Significance
	﻿Zhang et al., 2011 [66]	25(11/14)	﻿Life Satisfaction Index Z scale(Wood)	No Significance
	﻿Zhang et al., 2011 [66]	25(11/14)	﻿Self-esteem scale(Rosenberg)	Self-esteem
	﻿Caronni et al., 2017 [67]	402	﻿ ISYQOL	No Significance
	﻿Topalis et al., 2017 [68]	609(158/451)	﻿Self-assessment questionnaire	No Significance
	﻿Müller et al., 2010 [69]	2	﻿Interview	No Significance

*N*. (*C*/*E*) number of subjects in the control and experimental groups, *A* somehow affected, *NA* no affected, *BrQ* Brace Questionnaire, *BSSQ* Bad Sobernheim Stress Questionnaire, *SRS*-22 Scoliosis Research Society-22 Questionnaire, *SF*-36 The 36-item Short-Form, *SAQ* Spinal Appearance Questionnaires, *EQ*-5*D*-5*L* EuroQoL 5-dimension 5-level, *QLPSD* Quality of Life Profile for Spine Deformities, *SRS*-24 Scoliosis Research Society Instrument for Outcome Assessment 24, *GFS* General Function Score, *BFW* Berner Questionnaire for Well-Being, *CHQ* Child Health Questionnaire, *PODCI* Paediatric Outcomes Data Collection Instrument, *ISYQOL* Italian Spine Youth Qulaity of Life

## Discussion

This review classifies the literature based on the method used to measure the QoL and we found that the main affected life domains were self-image, mental health and vitality, which were separately discussed as below.

### Self-image

Law et al. [70] found that an aesthetically pleasing brace and the involvement of patients in the design process of the brace were important for increasing user compliance and also addressing psychological issues during treatment. Moreover, patients’ concerns on self-appearance inspired researchers to design flexible braces consisting of elastic straps and a soft shell, which allows more freedom of movement, less physical restrictions, and more importantly, allows to be hidden under clothes. To date, the most widely discussed flexible brace is SpineCor, which was proposed by the Sainte-Justine Hospital [71]. However, the effectiveness of SpineCor remains controversial. Guo et al. [72], Coillard et al. [73] and Wong et al. [74]. found significant differences between SpineCor and rigid brace group in terms of effectiveness. Whilst Gammon et al. [75] reported no significant difference in the treatment outcomes comparing thoraco-lumbar sacral orthosis (TLSO) and SpineCor-treated patients and Coillard et al. [76] demonstrated that SpineCor brace reduced the probability of the progression of early idiopathic scoliosis (15°–30°) after at least 5 years follow-up. However, patients’ acceptance and compliance (which have been shown to have a close correlation with the treatment efficacy [7, 77, 78]) to the SpineCor were comparable to rigid spinal orthoses. The SpineCor brace was also found to be better than TLSO at improving QoL, reported by Ersen et al. [37], patients treated with SpineCor brace have a better self-image, feel more active in daily life and experience less pain according to SRS-22 results. Whilst Misterska et al. [79] found that there was no significant difference in most of the analyzed domains of QoL between patients with the SpineCor brace and the Cheneau brace. Given the currently mixed outcomes of studies on flexible braces, we can conclude that even flexible braces, like SpineCor, has no comparable effectiveness as rigid brace, the merits of improving QoL are promising. A further challenge is in weighing potentially improved QoL against reduced effectiveness.

### Mental health

Mental health/psychological stress is defined as the distress AIS patients have because of their deformity or brace. Moreover, the impact of the brace to the self and body image of adolescent is reported as a contributing factor for stress production [80, 81]. This review has found that distress associated with bracing is significantly worse than distress associated with spinal deformity, based on the reviewed literature measuring psychological stress using BSSQ. Andersen et al. [82] found that uncertainty regarding the duration of the brace treatment is one of the reasons causing psychological sequela and they suggested a flexible bracing strategy, such as part-time bracing schemes where patients were urged to participate in sports and social activities without their braces, to avoid social isolation. Lin et al. [83] compared the stress levels of juvenile and adolescent idiopathic scoliosis patients with brace treatment and found that female adolescents were more vulnerable to depressive psychological status. Higher levels of cognitive function and independence and negative parental attitudes resulted in a greater incidence of depression.

### Vitality

Vitality is evaluated by patients’ feelings of energetic and enthusiastic attitudes to daily activities [19], which directly correlates to physical performance. Our findings that show a brace’s impact on vitality corroborate with Daryabor et al. [84], who reported a review on gait and energy consumption of AIS patients treated with orthoses. They found that after 6 months of treatment, excessive oxygen consumption was observed, and results of an endurance test also show a diminished exercise capacity caused by the brace. Moreover, a significant decrease in walking speed and more excessive energy cost were found from the subjects with AIS treated with orthoses versus those without orthoses. They suggested that it could be helpful to intensively train patients with endurance exercises to improve physical performance in AIS.

## Limitations

There are three limitations to this review: firstly, the methodology followed in this literature review treats all papers alike, regardless of potential quality differences, since this review aimed to capture the breadth of affected domains of QoL and to provide the results for informing future brace designs. Secondly, a risk of selection bias emerged since the results for RCTs (Randomized Controlled Trial) and non-RCTs are not separately presented to obtain more comprehensive results. RCTs would involve a direct comparison between braced and non-braced patients to provide more robust findings that non-RCTs. Thirdly, the most affected domains of QoL of patients with different severities of scoliosis have not been separated, and more specific details on the affected domains of QoL of patients wearing different braces and under different treatment stages also need to be evaluated.

## Conclusion

This paper presented a literature review on the impact of bracing on the Quality of Life of scoliotic adolescents. The results indicate that self-image, mental health, and vitality are the three most frequently reported affected domains. In order to improve the QoL of scoliotic brace wearers, these three domains should be prioritized in researching and designing new bracing treatment options.

## Footnotes

1 https://www.zotero.org/
